# Transition from Transrectal Systematic to Transperineal Lesion-Focused Prostate Biopsy: A Real-World Comparative Analysis

**DOI:** 10.3390/cancers18020332

**Published:** 2026-01-21

**Authors:** Thibaut Long Depaquit, Federica Sordelli, Christopher Agüero, Arthur Peyrottes, Alessandro Uleri, Laurent Daniel, David Chemouni, Cyrille Bastide, Michael Baboudjian

**Affiliations:** 1 Department of Urology, Nord Hospital, Assistance Publique–Hôpitaux de Marseille (AP-HM), 13005 Marseille, France; 2 Department of Urology, Saint-Louis Hospital, Assistance Publique–Hôpitaux de Paris (AP-HP), 75010 Paris, France; peyrottesarthur@gmail.com; 3 Department of Urology, Clinique de la Croix-du-Sud, 31130 Quint-Fonsegrives, France; alessandrouleri@outlook.it; 4 Department of Pathology, Nord Hospital, Assistance Publique–Hôpitaux de Marseille (AP-HM), 13005 Marseille, France; 5 Department of Radiology, Nord Hospital, Assistance Publique–Hôpitaux de Marseille (AP-HM), 13005 Marseille, France

**Keywords:** prostate cancer, targeted biopsy, regional biopsy, transperineal biopsy, PI-RADS

## Abstract

**Background/Objectives:** The transperineal (TP) approach has progressively replaced the transrectal (TR) approach for prostate biopsy because of its improved safety profile. However, its impact on the detection of clinically significant prostate cancer (csPCa), particularly within modern lesion-focused biopsy strategies that combine targeted and perilesional sampling, remains uncertain. We aimed to evaluate the real-world diagnostic impact of transitioning from a TR systematic-based biopsy strategy to a TP lesion-focused approach. **Methods:** We conducted a retrospective single-centre study including consecutive men who underwent image-guided prostate biopsy between 2018 and 2025. Only patients with a single MRI-visible lesion (PI-RADS ≥ 3) were included. Two biopsy strategies were compared: TR systematic biopsy (TR–SBx), combining targeted and systematic cores, and TP lesion-focused biopsy (TP–LFx), combining targeted and perilesional cores. The primary outcome was the detection of csPCa (Gleason Grade Group ≥ 2). Secondary outcomes included detection of Gleason Grade Group 1 cancer and negative biopsies. Inverse probability of treatment weighting (IPTW) based on a propensity score was applied to adjust for baseline differences. Doubly robust weighted logistic regression models were used, with predefined subgroup and sensitivity analyses. **Results:** Among 1032 included patients, 931 underwent TR–SBx and 101 TP–LFx. After restriction to the region of common support, 528 patients were retained for IPTW analyses. In the IPTW-adjusted analysis, TP–LFx was associated with higher csPCa detection compared with TR–SBx (adjusted odds ratio [OR] 2.52, 95% confidence interval [CI] 1.40–4.52; *p* = 0.002) and with lower detection of Gleason Grade Group 1 cancer (OR 0.50, 95% CI 0.27–0.92; *p* = 0.03). Subgroup analyses suggested a stronger association in patients with prior negative biopsy and in anterior or apical lesions. **Conclusions:** In routine clinical practice, transitioning from a transrectal systematic-based biopsy strategy to a transperineal lesion-focused approach was associated with improved detection of csPCa and reduced overdiagnosis. These findings support the consideration of transperineal, lesion-focused MRI-guided biopsy strategies in contemporary prostate cancer diagnostics.

## Simple Summary

Prostate biopsy is essential for diagnosing prostate cancer, but the optimal biopsy technique remains debated. In our centre, we transitioned from a traditional transrectal biopsy approach to a transperineal biopsy strategy focusing on MRI-visible lesions. In this real-world study, we show that this change improved the detection of clinically significant prostate cancer while reducing the diagnosis of low-risk disease. These findings suggest that modern transperineal biopsy strategies may improve diagnostic accuracy in routine clinical practice.

## 1. Introduction

Prostate cancer (PCa) diagnosis still relies on histological confirmation by prostate biopsy, which remains the gold standard for tumour grading according to the International Society of Urological Pathology (ISUP) score (i.e., the Gleason Grade (GG) Group) [1]. Multiparametric MRI (mpMRI) now underpins modern prostate biopsy by accurately localizing suspicious lesions and guiding targeted sampling. Evidence from randomized trials shows that MRI-targeted biopsy (MRI-TBx) improves the detection of clinically significant prostate cancer (csPCa) and limits overdiagnosis of insignificant disease (insPCa) relative to systematic biopsy (SBx) [2,3,4]. Accordingly, the EAU recommends MRI-TBx as the primary diagnostic strategy in men with MRI-visible lesions, with selective additional cores when necessary [5].

The transperineal (TP) route has become the preferred approach over the transrectal (TR), virtually eliminating infectious complications and supporting antibiotic stewardship. However, its impact on csPCa detection remains debated. Most comparative trials [6,7,8,9] used combined MRI-TBx + SBx templates, an approach now challenged by lesion-focused protocols integrating TBx and perilesional biopsies (PBx) while omitting distant cores [10,11,12]. Radiologic and pathologic studies show that MRI underestimates tumour boundaries and that most csPCa missed by targeted cores lie within approximately 10 mm of the lesion margin [13]. In line with these findings, recent EAU Guidelines acknowledge that perilesional sampling captures many of these missed Pca and may reduce the need for contralateral cores [14].

While robust comparative evidence exists, most studies were conducted under controlled conditions and biopsy designs that are not always representative of real-life practice. Moreover, data directly comparing different biopsy templates remain limited. As centres progressively move away from systematic cores and adopt targeted-plus-perilesional schemes, real-world evidence assessing these modern approaches has become increasingly necessary. Against this background, in 2023 our centre transitioned from a TR systematic-plus-targeted protocol to a TP targeted-plus-perilesional approach. This study assesses the diagnostic impact of that transition in real-world practice.

## 2. Methods

### 2.1. Study Design and Population

We conducted a retrospective analysis of a consecutive single-centre cohort of men who underwent MRI-guided prostate biopsy between January 2018 and January 2025. Eligible patients were ≥18 years old and referred for biopsy because of elevated PSA and/or abnormal digital rectal examination (DRE). Biopsy-naïve patients and those with a prior negative biopsy were included. Exclusion criteria were contraindications to MRI or biopsy, known metastatic disease, or missing essential baseline variables.

### 2.2. MRI Acquisition and Reporting

All patients underwent mpMRI on 1.5-T or 3-T scanners. Imaging and reporting were performed by experienced uro-radiologists in accordance with ESUR standards [14]. mpMRI included T2-weighted, diffusion-weighted, and dynamic contrast-enhanced sequences, and lesions were scored using PI-RADS v2.1 [15]. Only patients with a single MRI-visible lesion (PI-RADS ≥ 3) were included.

### 2.3. Biopsy Procedures

Biopsies were performed by experienced urologists using predefined institutional definitions and cognitive MRI–ultrasound fusion:

- Systematic biopsy (SBx): 12-core sampling according to a sextant-based template;
- Targeted biopsy (TBx): ≥3 cores per MRI-visible lesion, using TR or TP approaches and cognitive or software fusion;
- Perilesional biopsy (PBx): Cores obtained within 10 mm of the lesion margin, following Brisbane et al. [11].

Two biopsy strategies were used during the study period:

- Transrectal Systematic-Based Strategy (TR–SBx): MRI-TBx combined with SBx, used before January 2023;
- Transperineal Lesion-Focused Strategy (TP–LFx): MRI-TBx combined with PBx, implemented thereafter.

### 2.4. Pathological Assessment

All biopsy specimens were reviewed by one dedicated uropathologist (L.D.) according to the 2019 International Society of Urological Pathology (ISUP) grading system [1]. csPCa was defined as GG ≥ 2, whereas insPCa corresponded to GG 1.

### 2.5. Outcome Measures

The primary outcome was the detection of csPCa. Secondary outcomes were the detection of GG1 cancer and negative biopsies. Subgroup analyses were predefined according to PI-RADS category, lesion location (anterior, transition, peripheral), and prior negative biopsy status.

### 2.6. Statistical Analysis

Baseline covariates included age, PSA, prostate volume, PSA density, continuous PI-RADS score, maximum lesion diameter, digital rectal examination findings, and prior negative biopsy. Lesion location and PI-RADS categories were described at baseline but were not included in the propensity score, as they were subsequently explored in predefined subgroup analyses to avoid overadjustment. A propensity score was estimated using a logistic regression model and used to generate stabilized inverse probability of treatment weights (IPTWs). Weights were restricted to the region of common support (propensity score 0.05–0.95) and trimmed at the 1st and 99th percentiles. Covariate balance before and after weighting was assessed using standardized mean differences (SMDs), with values < 10% indicating adequate balance.

The primary analysis was conducted using a doubly robust IPTW-weighted logistic regression model, with biopsy strategy (TP-LFx vs. TR-SBx) as the exposure and csPCa detection as the outcome. Results are reported as adjusted odds ratios (ORs) with 95% confidence intervals (CIs). As a sensitivity analysis, a marginal IPTW-weighted model was also performed. Subgroup analyses were conducted using the same stabilized IPTWs, with separate weighted logistic regression models fitted within each subgroup. Fusion modality was not included in the models owing to collinearity with biopsy route. All statistical tests were two-sided, and statistical significance was defined as *p* < 0.05.

## 3. Results

### 3.1. Baseline Characteristics

A total of 1032 patients met the inclusion criteria, of whom 931 (90%) underwent TR–SBx and 101 (10%) underwent TP–LFx (Table 1) for a single MRI-visible lesion. Baseline characteristics differed between groups, particularly regarding prostate volume, PSA density, PI-RADS distribution, and prior biopsy status. After restriction to the region of common support of the propensity score (0.05–0.95), 528 patients were retained for IPTW analyses (459 TR–SBx and 69 TP–LFx). Following the application of stabilized IPTW and trimming at the 1st and 99th percentiles, all covariates included in the propensity score model achieved excellent balance, with absolute standardized mean differences below 10%, indicating effective mitigation of baseline differences (Table 2). Using stabilized IPTW, the effective weighted sample corresponded to 605 patients.

### 3.2. Outcomes

In the IPTW-adjusted primary analysis, TP–LFx was associated with a significantly higher detection rate of csPCa compared with TR–SBx (adjusted OR 2.52, 95% CI 1.40–4.52; *p* = 0.002) (Table 3). Conversely, TP–LFx was associated with a lower detection rate insPCa (adjusted OR 0.50, 95% CI 0.27–0.92; *p* = 0.03).

### 3.3. Subgroup Analyses

Predefined subgroup analyses were performed using stabilized IPTW with doubly robust weighted logistic regression (Table 4). The adjusted odds ratio for csPCa detection with TP–LFx was 1.66 (95% CI 0.95–2.89) in biopsy-naïve patients and 5.52 (95% CI 1.99–15.31) in patients with a prior negative biopsy. When stratified by MRI suspicion level, adjusted odds ratios were 1.50 (95% CI 0.83–2.70) for PI-RADS 4 lesions and 2.63 (95% CI 0.94–7.31) for PI-RADS 5 lesions. According to lesion location, the adjusted odds ratio was 2.05 (95% CI 0.90–4.69) for peripheral-zone lesions and 11.53 (95% CI 3.49–38.04) for anterior or transitional lesions.

### 3.4. Sensitivity Analysis

Sensitivity analyses focusing on the primary outcome were performed using an extended propensity score model incorporating lesion location and PI-RADS categories. After restriction to the region of common support and application of stabilized IPTW, covariate balance for MRI-related and anatomical variables was improved (Table S1). In this sensitivity analysis, the association between biopsy route and detection of clinically significant prostate cancer was attenuated and did not reach statistical significance (adjusted OR 1.57, 95% CI 0.85–2.90; *p* = 0.1) (Table S2).

## 4. Discussion

In this real-world, single-centre analysis, transitioning from a TR–SBx to a TP–LFx was associated with a clinically meaningful improvement in diagnostic yield. After IPTW adjustment, TP–LFx increased the detection of csPCa (adjusted OR 2.52, 95% CI 1.40–4.52; *p* = 0.002) while reducing the diagnosis of insPCa (OR 0.50, 95% CI 0.27–0.92; *p* = 0.03), supporting improved diagnostic precision rather than simple sampling inflation.

From an absolute perspective, csPCa was detected in 44% of men in the weighted TR–SBx cohort and in 59% in the weighted TP–LFx cohort. Large prospective MRI-based programmes provide useful reference points to interpret these rates, although direct comparisons are limited by differences in study design and patient selection. In the Göteborg-2 trial, an MRI-informed pathway relying on TBx alone reduced detection of insPCa while maintaining overall csPCa detection; however, a subset of csPCa was identified only by SBx in the reference arm [16]. More broadly, csPCa detection in contemporary MRI-positive cohorts is strongly driven by MRI suspicion level, with consistently high detection rates reported in PI-RADS 4–5 lesions [17]. Taken together, these data support that the absolute csPCa rates observed in our study are plausible within modern MRI-driven diagnostic pathways and help contextualize the incremental yield associated with optimized sampling geometry.

The first determinant of the improved diagnostic performance observed with the new biopsy pathway likely relates to the biopsy route itself. Randomized trials illustrate how biopsy design and procedural standardization influence route-specific performance. The PERFECT trial did not demonstrate non-inferiority of TP biopsy for csPCa detection, likely reflecting lower sampling density and greater operator variability in the TP arm [7]. In contrast, the larger TRANSLATE trial, which ensured balanced core numbers and standardized procedures, reported significantly higher csPCa detection with TP biopsy [8]. The PREVENT trial further underscored the major safety advantage of TP biopsy, showing a near-complete elimination of post-biopsy sepsis, although it was not powered for oncologic endpoints [6]. Taken together, these data suggest that differences between TP and TR biopsy are driven less by the route per se than by sampling geometry and procedural constraints.

From an anatomical perspective, the TP trajectory provides more direct access to anterior and apical regions, which are less reliably sampled using the TR route because of rectal wall angulation [8]. This advantage likely explains the higher detection of anterior csPCa reported in the meta-analysis by Uleri et al. and observed in our subgroup analyses [18]. Beyond diagnostic performance, the TP route offers a well-established safety benefit, with multiple trials and meta-analyses demonstrating a near-complete elimination of post-biopsy sepsis [19,20,21,22], findings that underpin current EAU recommendations favouring TP biopsy [14]. Together, these anatomical and safety considerations support TP as the preferred access within contemporary MRI-guided biopsy.

The second determinant of improved diagnostic performance relates to the biopsy template itself. In our centre, the historical TR protocol combined SBx and TBx cores, a strategy known to increase the detection of MRI-invisible, low-grade tumours and contribute to overdiagnosis [23,24]. In 2023, this approach was replaced by a TBx plus PBx sampling scheme. This change was informed by radiologic–pathologic correlation studies showing that mpMRI underestimates tumour extent by 5–10 mm. Most csPCas missed by targeted cores lie within a 10 mm perilesional margin [10]. These findings have prompted a gradual move away from routine systematic biopsy toward more streamlined sampling strategies, as reported in recent meta-analyses [11,25]. Consistent with this shift, our results show that combining TBx plus PBx sampling improves csPCa detection while reducing the identification of insPCas.

In terms of clinical implications, our data do not support the universal omission of SBx but may provide hypothesis-generating signals for selected scenarios. Large prospective MRI-based trials have shown that MRI-targeted strategies reduce detection of insPCa while maintaining overall csPCa detection, although a subset of csPCa may still be detected only by SBx in some settings [2,3,26]. Within a lesion-focused framework, omission of SBx may be considered in carefully selected men with a single MRI-visible lesion and concordant clinical and radiological risk profiles [27]. In contrast, adding SBx may remain reasonable in cases of clinical–radiological discordance, equivocal MRI findings, or suspected multifocal disease [26]. These considerations should be interpreted cautiously given the retrospective design and limited events in subgroups.

Despite these consistent findings, several limitations inherent to the study design should be acknowledged. Its retrospective before–after design may introduce temporal confounding, although IPTW and consistent sensitivity analyses mitigate this risk. Group sizes were unbalanced because the TP–LFx protocol was implemented recently. As a result, the TP cohort was relatively small, with a limited number of events. This reduced statistical power, particularly for subgroup analyses, notably in anterior or transitional and PI-RADS 5 lesions. This imbalance likely explains the attenuation of effect estimates observed in sensitivity analyses using more complex models. In this setting, the inclusion of MRI-related variables may have led to overadjustment and collinearity. Operator-related factors, including fusion modality, anesthetic conditions, and learning curves, were not captured. All biopsies in the present study were performed by a single urologist with extensive experience in MRI-guided prostate biopsy. This ensured procedural consistency. However, these results may not be directly generalizable to less experienced operators or trainees, who have historically been more exposed to the TR approach [28]. Operator experience and prior training background may influence biopsy performance, particularly during a transition in biopsy route. Structured training and supervision may therefore be necessary to ensure reproducible outcomes when implementing transperineal biopsy in routine practice.

Restricting inclusion to patients with a single MRI-visible lesion improved cohort homogeneity but limits generalizability to multifocal disease. Safety outcomes and patient-reported measures were not available, precluding direct comparison of tolerability between biopsy routes.

From a technical perspective, several transperineal biopsy techniques have been proposed to facilitate this transition. Simplified free-hand or fan-based transperineal approaches may represent more accessible options during the early learning phase compared with template-based techniques, which require additional equipment and setup time, as previously described [29]. However, comparative data assessing diagnostic performance and learning curves between these techniques remain limited, and further prospective studies are warranted.

Nevertheless, this study provides rare real-world evidence on the diagnostic impact of a combined change in biopsy route and sampling template.

## 5. Conclusions

In this real-world study, a combined transition in biopsy route and sampling template was associated with improved diagnostic performance. Moving from a transrectal systematic-based strategy to a transperineal lesion-focused approach increased the detection of clinically significant prostate cancer while reducing the diagnosis of insignificant disease. These findings support the adoption of contemporary MRI-guided biopsy pathways that may help optimize diagnostic precision in routine practice.

## Figures and Tables

**Table 1:** Baseline characteristics (unweighted).

Variables	Overall (*n* = 1032)	TR–SBx (*n* = 931)	TP–LFx (*n* = 101)	*p* Value
Age at biopsy, years	69 (63–75)	69 (63–75)	69 (64–74)	0.659
Prior negative biopsy, *n*	290 (28)	272 (29)	18 (18)	0.015
Baseline DRE				0.4
Normal	677 (66)	610 (66)	67 (66)	
Abnormal	353 (34)	321 (34)	34 (34)	
PSA, ng/mL	9.0 (6.2–13.6)	9.0 (6.2–13.7)	8.2 (6.2–12.2)	0.401
Prostate volume, mL	53 (40–75)	55 (40–80)	45 (31–60)	<0.001
PSA density	0.17 (0.11–0.26)	0.16 (0.11–0.25)	0.20 (0.14–0.29)	0.002
Maximum diameter of the index lesion, mm	13 (10–17)	13 (10–18)	13 (9.5–17)	0.634
PI-RADS category				<0.001
3	220 (21)	214 (23)	6 (6)	
4	477 (46)	470 (50)	65 (64)	
5	335 (33)	247 (27)	30 (30)	
Lesion location				0.7
Peripheral	890 (87)	817 (88)	73 (72)	
Transitional	66 (6)	54 (6)	12 (12)	
Anterior	76 (7)	60 (6)	16 (16)	

Data are presented as median (interquartile range, IQR) or number (percentage). TR–SBx = transrectal systematic biopsy; TP–LFx = transperineal lesion-focused biopsy; DRE= digital rectal examination; PSA = prostate-specific antigen; PI-RADS = Prostate Imaging Reporting and Data System.

**Table 2:** Baseline characteristics before and after IPTW weighting.

Variables	Before Matching			After Matching		
	TR–SBx *n* = 931	TP–LFx *n* = 101	SMD	TR–SBx *n* = 459	TP–LFx *n* = 69	SMD
Age, years	69 (63–75)	69 (63–75)	1.5	69 (63–75)	69 (63–75)	4.3
Prior negative biopsy, *n*	272 (29)	18 (18)	8.9	101 (22)	14 (20)	1.6
Abnormal DRE, *n*	321 (34)	34 (34)	2.5	146 (32)	22 (32)	4.5
PSA, ng/mL	9.0 (6–13)	9.0 (6–13)	6.2	9.0 (6–13)	9.0 (6–13)	4.3
Prostate volume, mL	45 (35–64)	50 (35–75)	23.8	45 (35–64)	50 (35–69)	4.0
PSA density	0.20 (0.12–0.30)	0.18 (0.11–0.29)	8.4	0.20 (0.12–0.30)	0.19 (0.11–0.29)	6.8
Maximum lesion diameter, mm	13 (10–18)	13 (10–18)	5.2	13 (10–18)	13 (10–17)	4.9

Data are presented as median (interquartile range, IQR) or number (percentage). Standardized mean differences (SMDs) are reported to assess covariate balance before and after IPTW; an SMD < 10% was considered indicative of adequate balance. TR–SBx = transrectal systematic biopsy; TP–LFx = transperineal lesion-focused biopsy; DRE = digital rectal examination; PSA = prostate-specific antigen; IPTW = inverse probability of treatment weighting.

**Table 3:** Prostate cancer detection after IPTW adjustment.

Outcome	TR–SBx (*n* = 459)	TP–LFx (*n* = 69)	Adjusted OR (95% CI)	*p*-Value
Clinically significant prostate cancer (i.e., GG group ≥ 2)	203 (44)	41 (59)	2.52 (1.40–4.52)	0.002
Insignificant prostate cancer (i.e., GG group 1)	121 (27)	9 (13)	0.50 (0.27–0.92)	0.03
Negative biopsy	135 (29)	19 (28)	0.79 (0.49–1.27)	0.3

Data are presented as median (interquartile range, IQR) or number (percentage). IPTW= inverse probability of treatment weighting; OR = odds ratio; CI = confidence interval; GG = Gleason Grade.

**Table 4:** Subgroup analysis of clinically significant prostate cancer detection.

Subgroup	TR–SBx	TP–LFx	Adjusted OR (95% CI)	*p*-Value
Biopsy history				
Biopsy-naïve	158 (43)	38 (52)	1.66 (0.95–2.89)	0.074
Prior negative biopsy	45 (26)	15 (65)	5.52 (1.99–15.31)	0.002
PI-RADS category				
PI-RADS 4	92 (39)	31 (48)	1.50 (0.83–2.70)	0.18
PI-RADS 5	93 (53)	20 (78)	2.63 (0.94–7.31)	0.065
Lesion location				
Peripheral zone	185 (40)	17 (55)	2.05 (0.90–4.69)	0.088
Anterior/transitional	10 (22)	24 (63)	11.53 (3.49–38.04)	<0.001

Data are presented as *n* (%). Percentages refer to the proportion of patients with clinically significant prostate cancer within each subgroup. Adjusted odds ratios were estimated using inverse probability of treatment–weighted (IPTW) logistic regression models. TR–SBx = transrectal systematic biopsy; TP–LFx = transperineal lesion-focused biopsy; PI-RADS = Prostate Imaging Reporting and Data System.

## Data Availability

The datasets generated and/or analyzed during the current study are not publicly available due to institutional data protection policies but are available from the corresponding author upon reasonable request.

## Supplementary Materials

The following supporting information can be downloaded at https://www.mdpi.com/article/10.3390/cancers18020332/s1. Table S1. Covariate balance before and after IPTW weighting in sensitivity analysis. Table S2. Sensitivity analysis of prostate cancer detection after propensity score adjustment.
