# Supplementary material for: Transition from Transrectal Systematic to Transperineal Lesion-Focused Prostate Biopsy: A Real-World Comparative Analysis
Source: Cancers (Basel). 2026 Jan 21;18(2):332. doi: 10.3390/cancers18020332 (PMC12838667; doi:10.3390/cancers18020332)
Supplement: Supplementary file 1 [file cancers-18-00332-s001.zip › Table_S1.pdf]

Table S1. Covariate balance before and after IPTW weighting in sensitivity analysis

Covariate	SMD Before IPTW (%)	SMD After IPTW (%)
**Age, *years***	<10	<10
**PSA, *ng/ml***	<10	<10
**Prostate volume, *mL***	>10	<10
**PSA density**	>10	<10
**Prior negative biopsy**	>10	<10
**Digital rectal examination**	<10	<10
**PI-RADS category**	>20	<10
**Lesion location**	>25	<10

Legend:

Standardized mean differences (SMDs) are reported before and after inverse probability of treatment weighting (IPTW).

DRE= digital rectal examination; PSA= prostate-specific antigen; PI-RADS = Prostate Imaging Reporting and Data System
