# Supplementary material for: Transition from Transrectal Systematic to Transperineal Lesion-Focused Prostate Biopsy: A Real-World Comparative Analysis
Source: Cancers (Basel). 2026 Jan 21;18(2):332. doi: 10.3390/cancers18020332 (PMC12838667; doi:10.3390/cancers18020332)
Supplement: Supplementary file 1 [file cancers-18-00332-s001.zip › Table_S2.pdf]

Table S2. Sensitivity analysis of prostate cancer detection after propensity score adjustment

Outcome	TR–SBx (n = 459)	TP–LFx (n = 69)	Adjusted OR (95% CI)	p-value
**Clinically significant prostate cancer (i.e., GG group ≥2)**	203 (44)	41 (59)	1.57 (0.85–2.90)	0.1
**Insignificant prostate cancer (i.e., GG group 1)**	121 (26)	9 (13)	0.63 (0.29–1.35)	0.2
**Negative biopsy**	135 (29)	19 (28)	0.88 (0.51–1.53)	0.6

Legend:

Data are presented as median (interquartile range, IQR) or number (percentage).

OR = Odds Ratio; CI = Confidence Interval; GG = Gleason Grade.
